# Time-Dependent Layer Formation Process on Quartz Bed Particles during the Fast Pyrolysis Process of Wood

**DOI:** 10.1021/acsomega.5c10470

**Published:** 2026-01-15

**Authors:** Ali Valizadeh, Fanfan Xu, Evert J. Leijenhorst, William Wolters, Bart Bemthuis, Erik Nilsson, Marcus Öhman

**Affiliations:** † Energy Engineering, Division of Energy Science, 407846 Luleå University of Technology, SE-971 87 Luleå, Sweden; ‡ 389127 BTG Biomass Technology Group BV, P.O. Box 835, 7500 AV Enschede, The Netherlands; § BTG-Bioliquids, 7545 PN Enschede, The Netherlands; ∥ Materials Science, Department of Engineering Sciences and Mathematics, 407846 Luleå University of Technology, SE-971 87 Luleå, Sweden

## Abstract

Understanding the characteristics and formation process of bed particle layers resulting from interactions between ash-forming matter and bed material during fast pyrolysis is crucial for optimizing fast pyrolysis bio-oil (FPBO) production. However, research on this topic remains limited. In this study, the evolution of the bed particle layers formed on quartz bed particles during fast pyrolysis of wood was investigated across bench-, pilot-, and industrial-scale units. Bed material samples with different exposure times were characterized using scanning electron microscopy coupled with energy-dispersive spectroscopy (SEM/EDS), and focused ion beam-SEM (FIB-SEM) to assess the morphology, elemental composition, and thickness of the bed particle layers. Overall, the time-dependent formation and characteristics of the quartz bed particle layers were similar to those reported for the fluidized-bed combustion of woody biomass. The key difference, however, was that the layers formed during fast pyrolysis were significantly thinner and contained less Ca. The bed particle layer formation began early in the process through the deposition of Ca-rich ash particles, primarily on convex surfaces, likely followed by solid–solid diffusion of Ca^2^^+^ into the quartz core, forming a Ca-silicate-rich inner layer. The inner layer developed later and more sparsely in concave regions, resulting in thinner layers. After approximately 1 day, an outer layer developed on the convex surfaces due to continued ash particle deposition, while a K–Si-rich inner-inner layer (likely composed of K-rich silicates and associated with gaseous alkali diffusion) formed primarily in concave regions. Over time, the bed particle layer thickness approached a limiting value of approximately 4 μm, likely due to reduced growth of the inner layer, which may be attributed to diminished inward transfer of Ca^2^^+^ as the diffusion distance increased.

## Graphical abstract

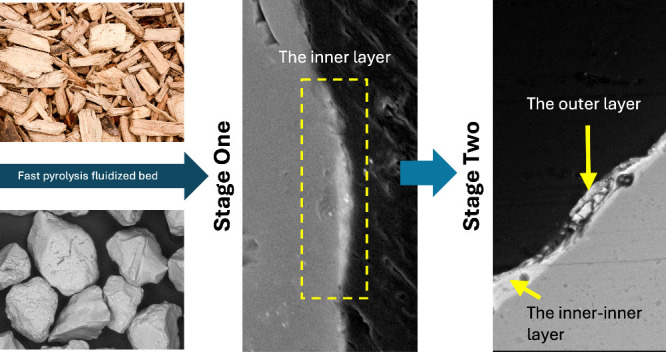

## 1 Introduction

The growing demand for sustainable and secure energy sources has positioned biomass as a key contributor to the global energy transition. As a widely available and renewable resource, biomass offers a viable alternative to fossil fuels, supporting both energy security and emissions reduction.^1–3^ Advancements in thermochemical conversion technologies have further unlocked its potential, enabling the efficient production of biofuels and high-value chemicals.^4^ Fast pyrolysis is a thermal decomposition process in which feedstock is rapidly heated to moderate temperatures (below 600 °C) in the absence of oxygen, resulting in the formation of condensable vapors, noncondensable gases, and solid char within a short residence time.^5^ The vapors are subsequently condensed into fast pyrolysis bio-oil (FPBO), which can then be upgraded to advanced biofuels through hydrodeoxygenation or converted into high-value chemicals via various catalytic processes.^6,7^ Several commercial biomass fast pyrolysis plants have been constructed in Europe and North America in recent decades, including facilities developed by BTG (BTG-BTL), VTT/Valmet, and Ensyn.^8,9^ These plants utilize a bed material that serves as a heat carrier, supplying the necessary thermal energy for the decomposition of biomass. The fuel is rapidly heated in a fluidized-bed or a rotating-cone pyrolysis reactor through direct contact with the hot bed material. The bed material and char are then separated from the produced vapors using a cyclone and are transported through a riser to the char combustor, where the char is fully combusted to reheat the bed particles. The reheated bed material is subsequently returned to the pyrolysis reactor. Natural sand, primarily composed of quartz, is commonly used as a heat carrier in large-scale plants due to its low cost and mechanical stability.^10^

Fast pyrolysis continues to attract considerable interest as a viable pathway for renewable fuel production owing to its ability to convert biomass into energy-dense liquid products in a compact and scalable system. Several recent studies have focused on improving reactor design, vapor residence time, and bio-oil upgrading strategies to enhance process efficiency and product quality.^11–15^ Additionally, pyrolysis-based systems are being increasingly recognized in policy and sustainability roadmaps for their potential to contribute to negative emissions when combined with biochar sequestration.^16^ These developments underscore the importance of addressing operational challenges, such as ash–bed material interactions, to ensure the long-term reliability and performance of commercial fast pyrolysis systems.

Although pyrolysis technology has advanced to the commercial stage, the process still requires ongoing optimization and refinement to maximize the yield and quality of FPBO and to mitigate potential operational risks.^17,18^ Feedstock pretreatment, operating parameters, reactor configuration, and biomass composition are important factors that influence FPBO production.^19,20^ For instance, it has been demonstrated that a higher ash content in the utilized biomass is associated with a reduced yield of bio-oil and an increased water content. This is due to the catalytic effects of inorganic species such as alkali and alkaline earth metals, which enhance cracking reactions, redirecting vapors toward gas and char formation and thus lowering FPBO yield.^21^ Moreover, fuel ash-derived compounds may promote secondary reactions among volatiles, further influencing char reactivity. Specifically, they increase the gas and char yield, reduce the liquid yield, and alter the distribution among these three products.^22^ It has been observed that alkali plays an important role in the secondary cracking reactions among volatiles during the pyrolysis process.^23^

In addition to the potential influence of the biomass ash-forming matter on the yield and composition of the products, interactions between the fuel ash-derived compounds and the bed particles in the fluidized-bed systems can also affect biomass thermochemical conversion processes. In particular, these interactions may lead to the formation of a layer on the surface of bed particles, resulting in bed agglomeration and material deposition.^24,25^ Such interactions have been extensively studied in the context of fluidized-bed combustion and gasification of woody biomass, particularly with respect to bed particle layer formation^26–29^ and the bed agglomeration mechanism.^30–33^ In fluidized-bed gasification of woody biomass, it has been demonstrated that the bed particle layers positively influence the process by enhancing the water-gas shift reaction and the steam reforming of tar.^34,35^ However, the potential catalytic activity of the bed particle layers is undesirable in fast pyrolysis, as it can negatively affect the yield and composition of FPBO.^36,37^ Although quartz is typically considered inert, there is ongoing debate in the literature about its potential to become catalytically active over time due to the accumulation of Ca- and K-rich ash layers on its surface. These transformations may not only reduce FPBO yield but also influence the selectivity of pyrolysis vapors by promoting secondary reactions such as cracking or reforming.^36–39^ This underscores the importance of understanding how bed particle layers evolve under realistic process conditions. While the catalytic significance of these layers in gasification and their operational impacts in both combustion and gasification are well-recognized, far less attention has been given to their formation kinetics in fast pyrolysis—specifically, how rapidly Ca- and K-rich phases accumulate on the bed particle surface and how this time-dependent evolution influences vapor-phase reactions, FPBO quality, and long-term reactor stability.

When quartz is used as the bed material in fluidized-bed combustion of woody biomass, multiple layers with varying properties and compositions are often found on the bed particles.^34^ The inner layer, also known as the Ca-reaction layer, is relatively homogeneous and forms due to the reaction between fuel-derived Ca compounds and the bed material. On the other hand, the outer layer, also referred to as the deposition layer, forms from the deposition of fine fuel ash-derived particles (e.g., CaO), typically smaller than 10 μm, resulting in a composition that closely mirrors that of the fuel ash.^34,35,40^ For bed particle layer formation in fluidized-bed combustion of woody biomass, in which the temperature is high enough to release K to the gas phase, primarily in the form of KCl and KOH, it has been proposed that the onset of the layer formation process is driven by low-melting K-silicates formed due to the reaction of gaseous K compounds with quartz bed particles.^41^ Once the initial melt forms on the surfaces of the bed particles, ash-derived Ca-rich particles adhere to this sticky layer, subsequently dissolving and reacting with the silicate melts. In the early stages of deposition, the chemical nature of the quartz surface may also influence the ash particle attachment. Specifically, surface hydroxyl groups (silanols, Si–OH) present on quartz have been shown to act as active adsorption sites for divalent cations such as Ca^2^^+^. Molecular dynamics simulations have demonstrated that these groups can facilitate ion binding at the quartz–ash interface, potentially promoting initial layer nucleation on fresh bed particles under fluidized-bed conditions.^42^ The Ca^2^^+^ from the deposited Ca-rich ash particles diffuses into the melt and replaces K, whereby the amount of melt decreases and crystalline Ca-silicates form, which starts to constitute the inner layer. Upon further diffusion of Ca^2^^+^ from the outer layer, the formation process of the inner layer becomes diffusion-controlled, and Ca-silicates with a higher number of Ca in their structure will form, resulting in the formation of cracks in the inner layer due to lattice mismatch.^41^ Additionally, studies have shown that in regions where the bed layer is absent or very thin, particularly in concave areas and also through the cracks in the inner layer, gaseous alkali can infiltrate the bed particle core and react to form K-silicates. This region is commonly termed the inner-inner layer (adjacent to the bed particle surface) or the crack layer (extending toward the particle core). Due to its formation mechanism, this region is also described as the K-reaction layer in the literature.^29,41,43^

In a previous study, the bed particle layer characteristics on quartz particles used in three different industrial-scale fast pyrolysis plants for woody biomass were compared to those observed in the combustion processes with similar exposure times. The results showed that the layers were significantly thinner in the fast pyrolysis plants, and the concentration of Ca was lower compared to those in the combustion.^10^

At elevated temperatures, formation of sticky K-silicate melts improves the adhesion of the ash-derived particles upon impact on the bed particle surface.^44^ Additionally, the diffusion of Ca^2^^+^ into the inner layer of bed particles is enhanced at higher temperatures due to increased mass diffusivity.^45^ Furthermore, the reaction kinetics of Ca–Si interactions are also accelerated at elevated temperatures.^46^ In the commercial fast pyrolysis processes, the bed temperature is lower (approximately 500 °C in the pyrolysis reactor and around 650 °C in the fluidized-bed combustion reactor) compared to the bed temperatures in fluidized-bed combustion, which range from 750 to 900 °C.^10,47^ Consequently, the bed particle layer formation process may differ between the fast pyrolysis and combustion of wood due to the lower operating temperature.

The interactions between quartz bed particles and the main ash-forming elements in fluidized-bed combustion of woody biomass, along with the time-dependent bed particle layer formation in these processes, are well-documented in the literature.^29,41,43,48,49^ Previous studies have extensively described the formation of Ca- and K-rich layers on quartz bed particles under fluidized-bed combustion conditions, where high temperatures (750–900 °C) promote alkali volatilization, rapid formation of K-rich silicate melts, and accelerated Ca–Si diffusion.^41,43^ In a previous study, it was demonstrated that these conditions lead to pronounced morphology-dependent layer development, with convex regions accumulating thicker layers than concave regions.^50^ However, it remains unclear whether such morphology-controlled mechanisms persist under fast pyrolysis conditions, where the volatilization of K is less pronounced than in combustion and Ca release is delayed until char combustion. Moreover, recent work in industrial fast pyrolysis plants has shown that the resulting quartz bed particle layers are significantly thinner and less Ca-rich compared to those formed in combustion;^10^ however, these findings were based solely on single-time-point industrial samples, without resolving the temporal development of the layers.

Given the operational importance of bed particle layers in fast pyrolysis, particularly their implications for ash–bed interactions, bed material circulation, and long-term reactor reliability, there is a clear need for a mechanistic, time-resolved understanding of how these layers develop under pyrolysis-relevant conditions. Accordingly, the primary objective of this work is to elucidate the formation, morphology, composition, and time-dependent evolution of bed layers on quartz bed particles during the fast pyrolysis of woody biomass.

Addressing this knowledge gap is particularly important, given the increasing interest in fast pyrolysis as a platform for producing renewable fuels and achieving large-scale carbon reductions. The present work addresses these knowledge gaps by providing the first time-resolved, multiscale (bench-, pilot-, and industrial-scale) investigation of bed particle layer formation under fast pyrolysis conditions. In doing so, the study examines the time-dependent evolution of these layers on quartz particles and compares the observed mechanisms to those previously reported for fluidized-bed combustion of wood. The findings from this work contribute to a deeper understanding of bed particle layer formation and will support efforts to mitigate ash-related challenges, thereby advancing the commercialization of the fast pyrolysis process.

## 2 Materials and Methods

### 2.1 Bed Material Sampling Campaigns

The fast pyrolysis experiments were conducted in bench-scale, pilot-scale, and industrial-scale fast pyrolysis units utilizing rotating-cone technology. In all cases, commercially sourced quartz sand was used as the bed material without any surface pretreatment, consistent with the standard practice in fluidized-bed systems. The particles were dry-sieved to the target size range before use. Simplified process flow diagrams for these processes are shown in Figures 1 and 2. The studied fast pyrolysis system comprised a rotating-cone pyrolysis reactor, a riser, a bubbling fluidized-bed char combustor, and a downcomer. The biomass feedstock was transported to the reactor through the feeder (in the bench- and pilot-scale units) or the dosing system (in the full-scale unit). In the rotating-cone pyrolysis reactor, biomass particles and hot bed particles were thoroughly mixed in the absence of oxygen, resulting in the release of organic vapors. For bench-scale and pilot-scale units (Figure 1), the produced vapors passed through cyclones and subsequently entered the condenser, where they were quenched by recirculated oil to produce the FPBO and the pyrolysis gas. Meanwhile, the char separated from the vapors was redirected to the combustor via the riser along with the bed material from the downcomer. In the bubbling fluidized-bed combustor, char was burned with air to reheat sand particles, which were then returned to the pyrolysis reactor to provide the required heat for the fast pyrolysis process. After passing through a filter, the flue gas and fly ash generated from the char combustion were separated. For the full-scale plant (Figure 2), the produced vapors, the bed material, and the char converged from the downcomer into a separator, followed by cyclones for gas–solid separation. The vapors were directed to the condenser, where they were cooled with the recirculated oil to collect the FPBO, while the noncondensable pyrolysis gases were transported into the freeboard section of the combustor. In the bubbling fluidized-bed combustion reactor, char and bed material from separation were completely burned to reheat the bed particles, which were then sent back to the pyrolysis reactor through a sand cooler to control the bed temperature. During the bed material circulation, approximately 90% of the bed remained in the bubbling fluidized-bed combustor, while the remainder was directed to other sections such as the riser, the sand cooler, and the rotating core pyrolysis reactor.

**Figure 1:**
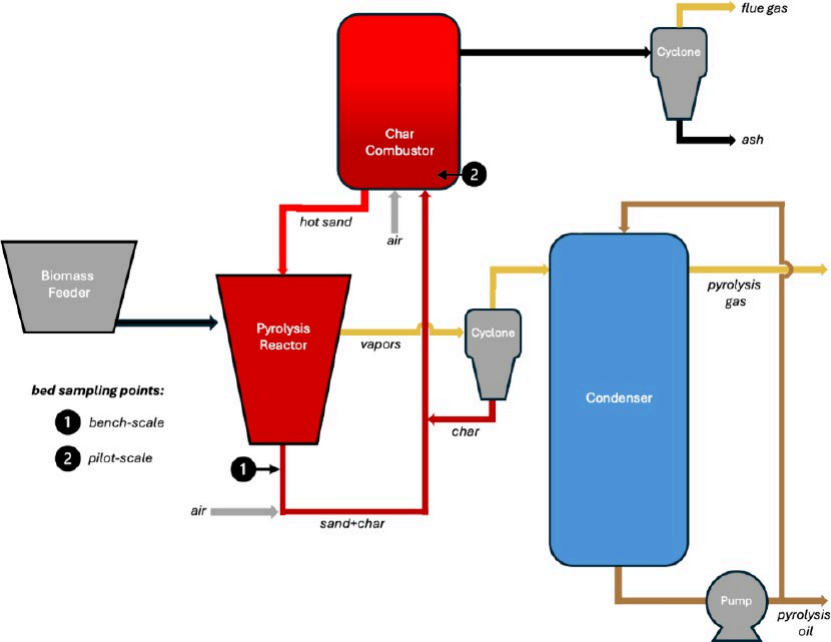
Process flow diagram of the bench-scale and pilot-scale fast pyrolysis system based on rotating-cone technology.

**Figure 2:**
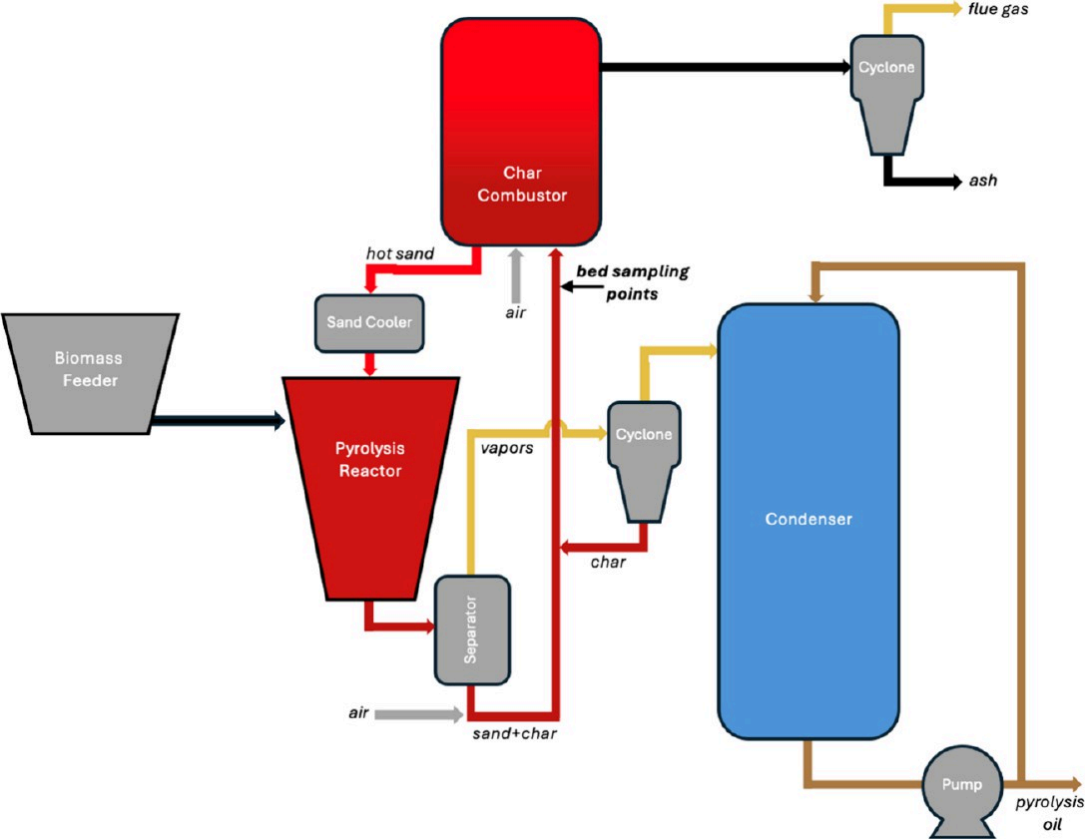
Process flow diagram of the industrial-scale fast pyrolysis system based on rotating-cone technology.

No sulfur sorbents (e.g., limestone) or in situ desulfurization additives were used in any of the experiments. Therefore, any Ca interacting with the bed material during operation originates solely from the inherent ash-forming matter of the woody fuel.

The fuel ash analysis for the biomass feedstock and the operating conditions under which the experimental campaign was conducted for the three studied units are presented in Tables 1 and 2.

**Table 1:** Ash Content and Main Ash-Forming Elements of the Biomass Feedstocks Used in Bench-, Pilot-, and Full-Scale Bed sampling Campaigns

	bench-scale/pilot-scale	full-scale^10^
Biomass fuels	pine sawdust	powder from stem-wood pellets
Average particle size (mm)	1.87	1.10
Ash content (wt %, d.b.^a^)	0.20	0.59
Main ash-forming elements (wt% d.b.)		
K	0.0443	0.0927
Na	0.0007	0.0131
Ca	0.0903	0.2052
Mg	0.0179	0.0359
Si	0.0090	0.0896
Al	not analyzed	0.0115
P	0.0030	0.0665
S	0.0086	0.0258
Cl	0.0174	0.0135
Mn	0.0065	0.0173
Fe	0.0023	0.0187

a Dry basis. Analysis with ICP-SFMS according to SS-EN ISO 17294-1, 2, and EPA method 200.8.

**Table 2:** Summary of the Bed and Operational Data for the Different Bed Sampling Campaigns

	unit	bench-scale	pilot-scale	full-scale^10^
pyrolysis reactor bed temperature	°C	500	∼500	∼500
combustion reactor bed temperature	°C	580	∼650	∼650
biomass feeding rate	kg h^–1^	4.6	136	5000
bed initial mass	kg	15–20	500	20,000
sand circulation rate	kg h^–1^	60	2500	10,000
biomass-to-sand circulation ratio	kg sand/kg biomass	12	18	20
biomass feed rate to initial bed mass	kg biomass/h per kg sand	1:4	1:4	1:4
average bed particle size	μm	285	285	420
bed sampling time from startup	h	4, 8, 12, 16, 18, and 22	96	∼1000
sampling position		after the pyrolysis reactor	bottom of the combustion reactor	bottom of the combustion reactor

#### 2.1.1 Bed Material Sampling

For the bench-scale unit, the bed samples were taken from the loop seal of the downcomer after the pyrolysis reactor (marked as location 1 in Figure 1) during steady-state operation at intervals of 4, 8, 12, 16, 20, and 22 h from the startup with a fresh bed. These sampling intervals were selected based on prior experience and practical considerations, aiming to capture the onset and progression of layer formation where detectable changes were expected. Earlier time points were not included, as any initial deposits at that stage were anticipated to be below the detection limit of SEM and of limited relevance for industrial conditions, where measurable layer development becomes operationally significant. For the pilot-scale unit, the bed samples were collected from the bottom of the combustor (marked as location 2 in Figure 1) after 96 h of operation following the startup with a fresh bed. No fresh bed material was added during the process for the bench-scale and pilot-scale runs.

In the industrial-scale unit, bed samples were collected from the bottom of the combustor (marked in Figure 2) after 2 months of operation (∼1000 h). The initial bed inventory was 20 tons, and throughout the experimental campaign, additional fresh bed material was added monthly at a rate of 5 tons to compensate for particles lost due to fragmentation.

By combining the results from the various time intervals of the bench-scale process with those from the pilot- and industrial-scale processes, a more holistic understanding of the bed particle layer formation process is achieved. This approach allows for a thorough examination of the progression from the early stages of layer development to the formation of fully developed bed particle layers, providing a clearer picture of how the process evolves over time across different scales.

### 2.2 Bed Particle Layer Characterization

Two distinct sets of SEM images were acquired to comprehensively characterize the particle layers. The first set focused on the surface of the bed particles to examine the formation and distribution of the layers at different stages of the process. These surface images provided insight into the extent and uniformity of the layer development over time. The second set consisted of cross-sectional images obtained from particles embedded in epoxy resin that were sectioned to reveal the internal structure. These images enabled detailed observation of the layer morphology and allowed for accurate measurement of the layer thickness, offering a clearer understanding of its temporal evolution.

Prior to SEM analysis of the cross sections, the bed particles were dry-sieved. Furthermore, to achieve high-resolution imaging of the extremely thin layer formed on the younger bed particles (<20 h), cross sections embedded in epoxy resin were coated with a 20 nm layer of platinum. The bed particle samples were analyzed with a JSM-IT300 (JEOL, Japan) SEM apparatus equipped with an X-Max 80 EDS detector (Oxford Instruments, UK). A backscattered electron detector (BSE) was employed in the scanning to produce compositional and phase contrast.^51^ This was essential for accurately distinguishing between the bed material and different layers within the analyzed bed particle samples. The SEM analyses were conducted in low vacuum mode (100 Pa) at a working distance of 10–12 mm. To reduce the electron beam penetration and minimize the interference from the core of the bed particles in the EDS measurements, an accelerating voltage of 10 kV was selected. While this setting limits the interaction volume to approximately 1–2 μm, some signal contribution from the substrate is still possible in very thin outer layers. Therefore, in the quantification of elemental composition, the Si signal was excluded to avoid artificial inflation of its value due to electron beam penetration into the quartz core, which is particularly significant in ultrathin surface layers.

For the cross-sectional analysis, 10 typical layered bed particles were selected to ensure representative results. To minimize the influence of noncentral cross sections, only particles with cross sections closely matching the average diameter of the selected size fraction (200–300 μm) were used to reduce the risk of including noncentral sections. Among these, only those exhibiting the thickest layers were selected for analysis. This approach not only enhanced the reliability of both the morphological evaluation and the measurement of layer thickness but also effectively excluded the influence of newly added particles from periodic bed material replenishment in an industrial-scale process.

The elemental composition of the layers was analyzed on a C- and O-free basis using point analysis mode at approximately 30 locations evenly distributed across the surface of each analyzed bed particle. These spots were selected in both the convex and concave areas of the particles to compare the layer characteristics between these regions.

To accurately assess the thickness of the bed particle layers, an image analysis procedure was applied to the cross-sectional SEM images of the selected bed particles. The total thickness of the bed particle layers (including both inner and outer layers, if present) was then quantified at more than 100 equidistant points around each bed particle’s cross section. The measurements were taken only in areas where a layer was present, as some parts of the bed surface, particularly in the younger bed samples, did not exhibit any layer formation. Therefore, uncoated (zero-thickness) areas were not included in the averaging. Further information on this procedure can be found elsewhere.^50^

To complement the SEM-EDS analyses and further investigate the ultrathin layers formed during the initial stages of the process, focused ion beam scanning electron microscopy (FIB-SEM) was also employed. The FIB-SEM analyses were performed by using an Amber X2 FIB-SEM system (TESCAN, Czech Republic). To achieve superior spatial resolution, SEM imaging was conducted at 20 keV and 300 pA, using an ultrahigh (UH)-resolution setting. The objective was to verify the presence of a distinct surface layer, potentially enriched in calcium, similar to the previously reported observations on quartz bed particles. For this purpose, FIB-SEM was applied selectively to convex areas of the 12 h samples, where early-stage layer formation was clearly visible in the cross-sectional SEM images. This analysis was intended to complement the broader SEM-EDS evaluation across time points. A lift-out sample was extracted from a convex region of the particle to enhance the likelihood of capturing any layer development. This cross section was then subjected to high-resolution SEM imaging and EDS analysis.

It should be mentioned that due to the very small thickness of the bed particle layers relative to the quartz substrate, bulk analytical techniques such as XRD or XRF were not suitable for phase identification and elemental analysis, respectively, as the signals would be dominated by the underlying SiO_2_ matrix. Therefore, SEM/EDS and FIB-SEM were employed as appropriate techniques for spatially resolved characterization of the layer composition and structure.

## 3 Results and Discussion

### 3.1 Bed Particle Layer Morphology and Composition

Figure 3 presents SEM images of the surface of typical quartz bed particles taken at different time intervals from the bench-scale process. Figure 4 displays SEM images of the surface of quartz bed particles taken at the end of the campaign from the pilot-scale and industrial-scale processes. As could be observed from the figures, the formation of the bed particle layer could be observed as brighter areas, mainly on the convex regions of the quartz bed particle surfaces. For the younger bed particles, these brighter areas appeared as isolated spots (Figure 3). However, as time progressed, the spots became more continuous, gradually forming a more uniform layer. The development of the bed particle layers was observed in the quartz bed particle samples taken from the longer exposures (i.e., the pilot scale and industrial scale), where the bed particle layer covered a larger portion of the bed particle surface rather than being confined to the convex areas, as observed in the bench-scale samples (Figure 4). However, the overall observation was that the fine fuel-derived ash particles found on the bed particles taken from the pilot-scale process were significantly less sintered (Figure 4a_2_) compared to those from the industrial-scale process (Figure 4b_2_), likely due to the shorter exposure time in the process. Notably, in the concave areas of the industrial-scale bed particles, nonsintered fine fuel-derived ash particles were also observed. This may be attributed to the effects of attrition, which predominantly impact convex surfaces that are more exposed to mechanical interactions.

**Figure 3:**
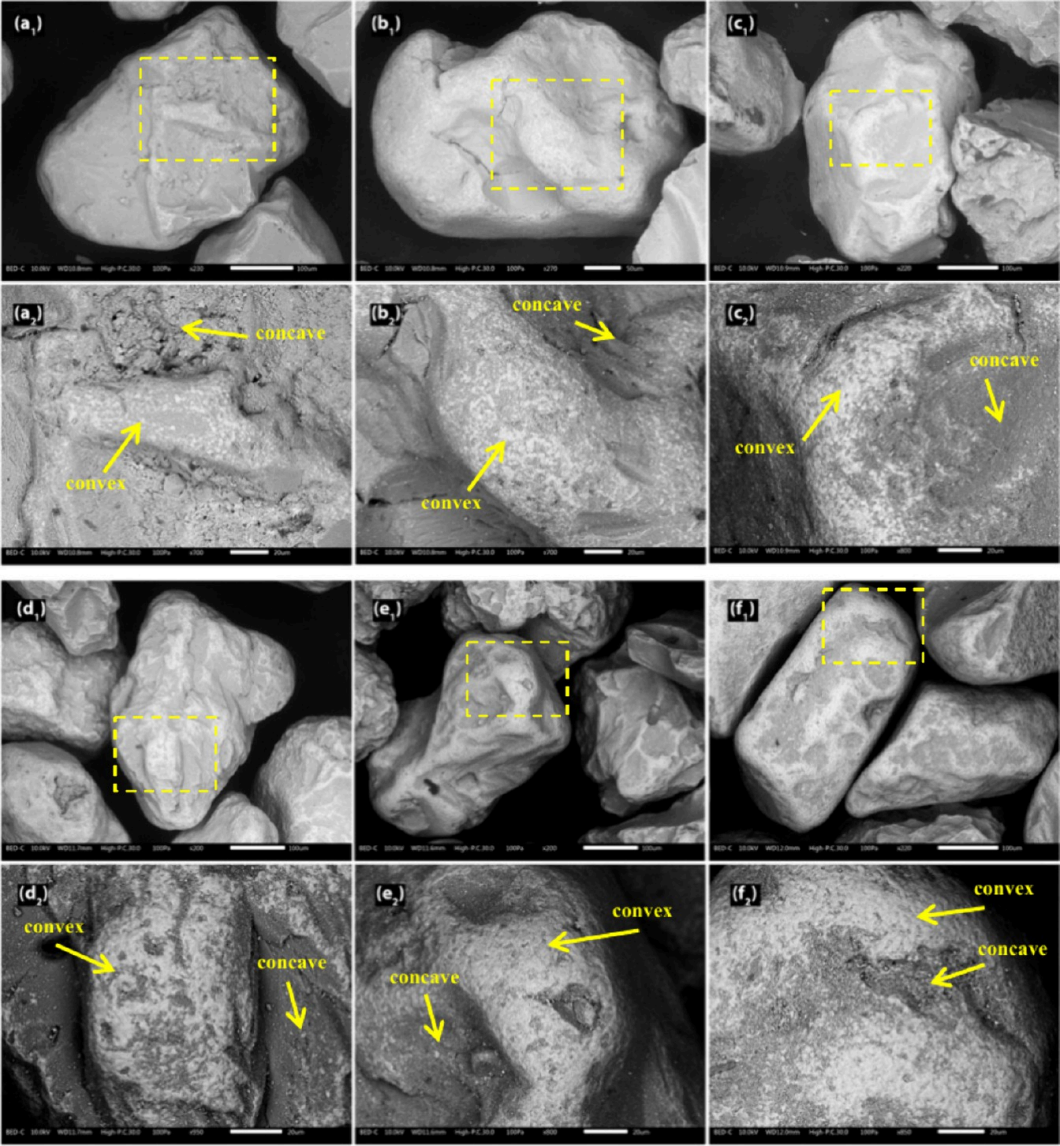
Surface SEM-BSE images of the typical quartz bed particles taken after (a1) 4 h, (b1) 8 h, (c1) 12 h, (d1) 16 h, (e1) 20 h, and (f1) 22 h from the bench-scale process. The yellow dashed rectangles indicate the areas that are magnified in panels (a2) to (f2), showing detailed surface features of the corresponding image.

**Figure 4:**
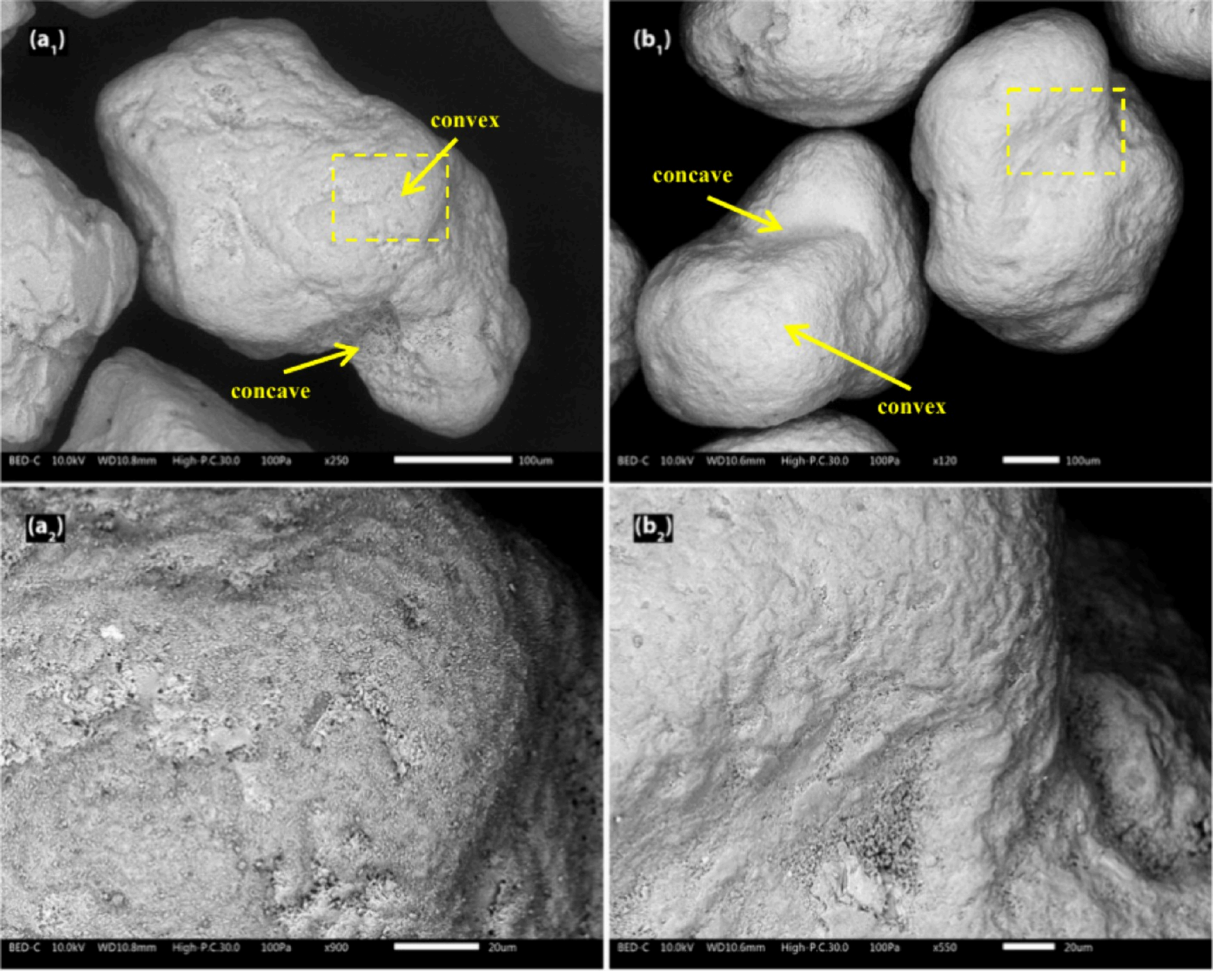
Surface SEM-BSE images of typical quartz bed particles taken at the end of the experimental campaign for (a1) the pilot scale (96 h) and (b1) the industrial scale (∼1000 h). The yellow dashed rectangles indicate the areas that are magnified in panels (a2) to (b2), showing detailed surface features of the corresponding image.

To further support the formation of the bed particle layer, EDS analysis was conducted at different spots on the bed particles’ surface to detect the composition and compare the results with previous observations of bed particle layer formation on quartz bed particles, documented in the literature.^10,43,48,49^ The average elemental composition on the bed particle surface obtained from EDS spot analysis is presented in Figure 5. Due to the very thin nature of the formed layers, it is likely that the electron beam penetrated through the surface deposit and interacted with the underlying quartz substrate. As a result, a considerable portion of the detected Si signal may not represent the surface layer itself but rather originate from the bed particle core. To avoid misinterpretation of the surface composition, Si was therefore excluded from the reported EDS results. It could be observed that the bed particle surface was primarily dominated by Ca and Mg (except O), which is consistent with previous observations.^10^ The notable concentration of Al could be attributed to feldspar impurities in the bed particles, as the natural sand contained small fractions of this mineral.

**Figure 5:**
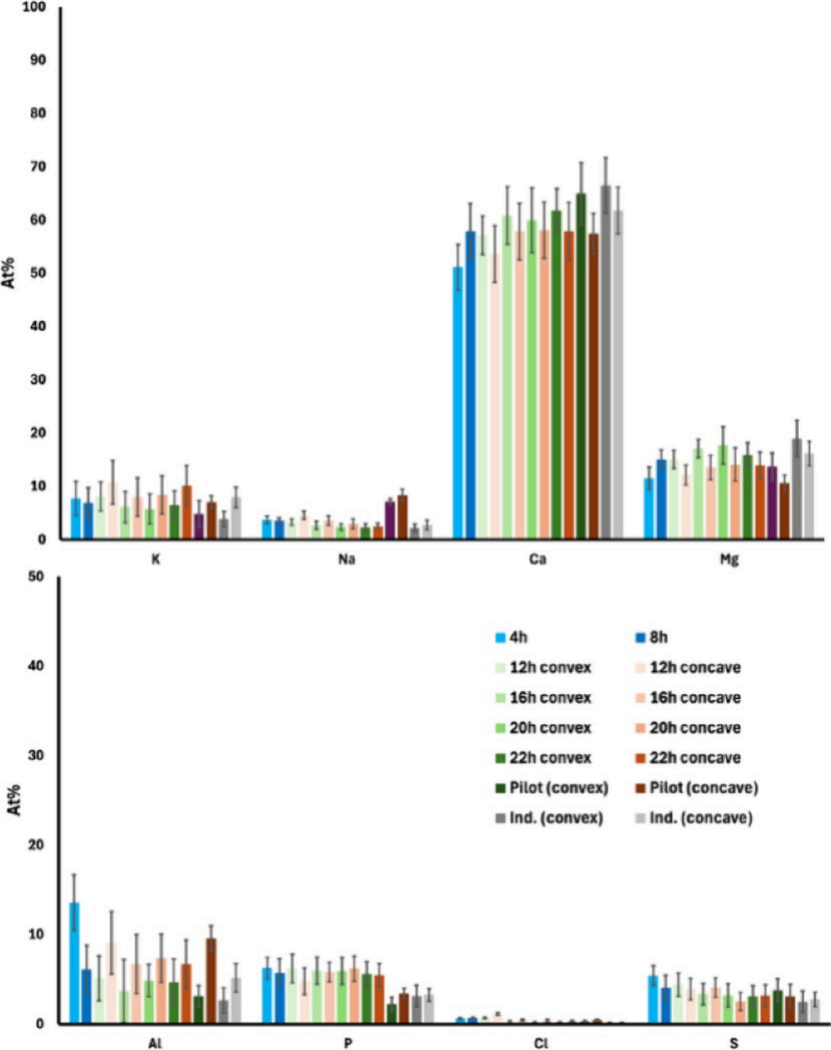
Average elemental composition (on a Si-, C-, and O-free basis) of the bed particle surface for the typical quartz bed particles collected from the bench-scale, pilot-scale (96 h), and industrial-scale (∼1000 h) processes. The error bars show the standard deviation at a 95% confidence interval.

Although the bed particle layer formation was observed in the convex areas starting at 4 h, it was not detected in the concave areas until 12 h, indicating a delayed development of the bed particle layer formation in these regions.

Figure 6 shows cross-sectional SEM images of typical quartz bed particles taken from the bench-scale process at different time intervals. Due to the bed particle layers being very thin and initially forming as sporadic spots on convex areas, they were not visible in the cross-sectional images for the 4 h and 8 h samples. After 12 and 16 h, a single, homogeneous layer, visually resembling the inner layer previously reported in the literature, was observed on the convex regions of the bed particles.^10,41^ For the 20 h and 22 h samples, the outer layer appeared in certain areas as a heterogeneous layer, composed of very fine ash particles and showing morphological features consistent with earlier observations reported in the literature. However, due to the very thin nature of the layers, a precise distinction of the borderline between the inner and outer layers was not possible. Furthermore, in some concave areas of the samples taken after 22 h, an inner-inner layer was also detected, in line with previous observations for quartz bed particles and characterized by a composition dominated by Si, K, and Ca.^10,29^

**Figure 6:**
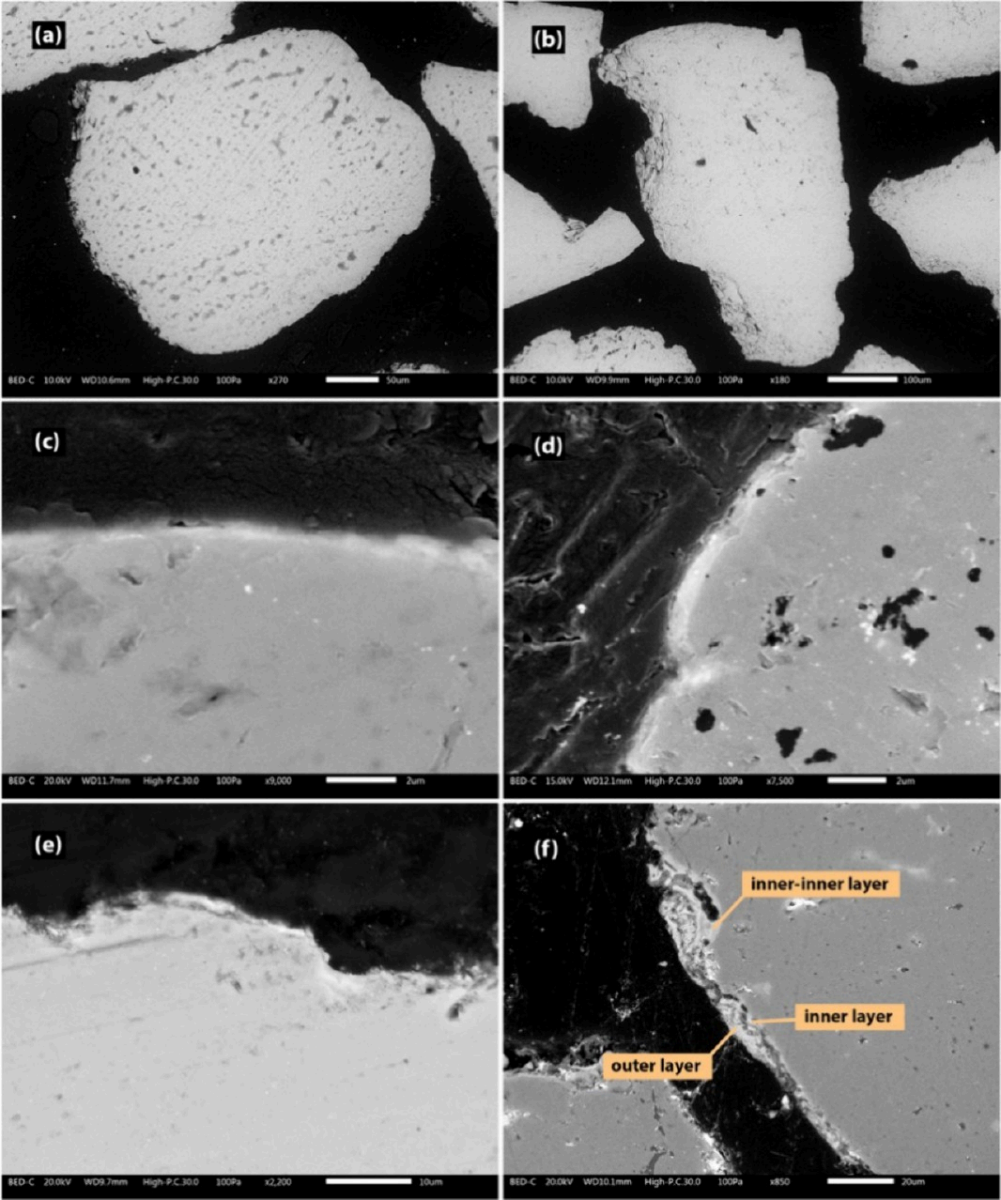
Cross-sectional SEM-BSE images of typical quartz bed particles taken after 4 h (a), 8 h (b), 12 h (c), 16 h (d), 20 h (e), and 22 (f) h from the bench-scale process.

None of the observed layers on the quartz bed particles collected from the bench-scale process covered the entire periphery of the bed particle; instead, they were sporadically scattered around the surface, primarily in the convex regions.

EDS analysis of the individual layers in the cross-sectional views was not feasible for the samples collected from the bench-scale process, as the layers were much thinner than the technique’s spatial resolution. Consequently, the detected signals included contributions from the quartz core, making it unreliable to distinguish the composition of each layer. However, the elemental profiles consistently indicated a dominance of Si, Ca, and Mg within the formed inner layer.

To overcome these limitations and verify whether the observed features were genuine layers or imaging artifacts, focused ion beam scanning electron microscopy (FIB-SEM) was employed for higher-resolution analysis. As shown in Figure 7, the analysis confirmed that the layer retains higher concentrations of Ca and Mg, with a lower Si content than the underlying bed particle core, which exhibits a higher Si content, although with lower amounts in the areas directly beneath the periphery. Additionally, it is evident from the image that Si is present in areas with Ca, suggesting the formation of a reaction layer, likely calcium silicate. This supports the hypothesis that the lighter periphery is indeed a distinct bed particle layer, similar to the inner layer observed in previous findings in the literature, rather than being an imaging artifact.

**Figure 7:**
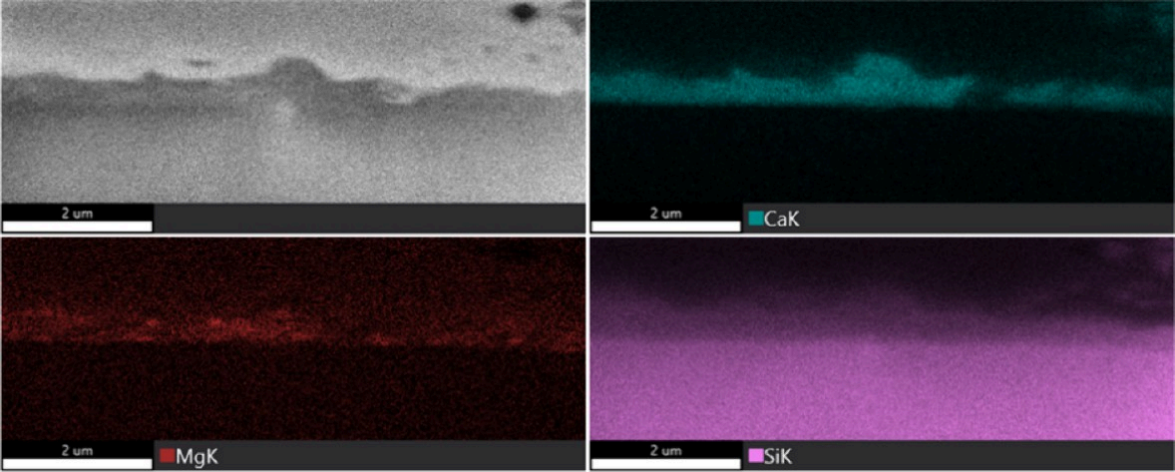
FIB-SEM image of a typical quartz bed particle taken after 12 h from the bench-scale process together with the EDS elemental mapping of Ca, Mg, and Si.

Figure 8 shows cross sections of typical quartz bed particles taken at the end of the campaign from both the pilot- and industrial-scale processes. The bed particle layer covers the majority of the bed particle periphery, with the coverage being even more pronounced in the samples from the industrial-scale process. In Figure 9, the bed particle layers formed on typical quartz bed particles collected from both the pilot- and industrial-scale processes are presented in more detail. It could be seen that at longer exposure times, different bed particle layers (the inner layer, outer layer, and inner-inner layer) become clearly visible around the bed particle periphery.

**Figure 8:**
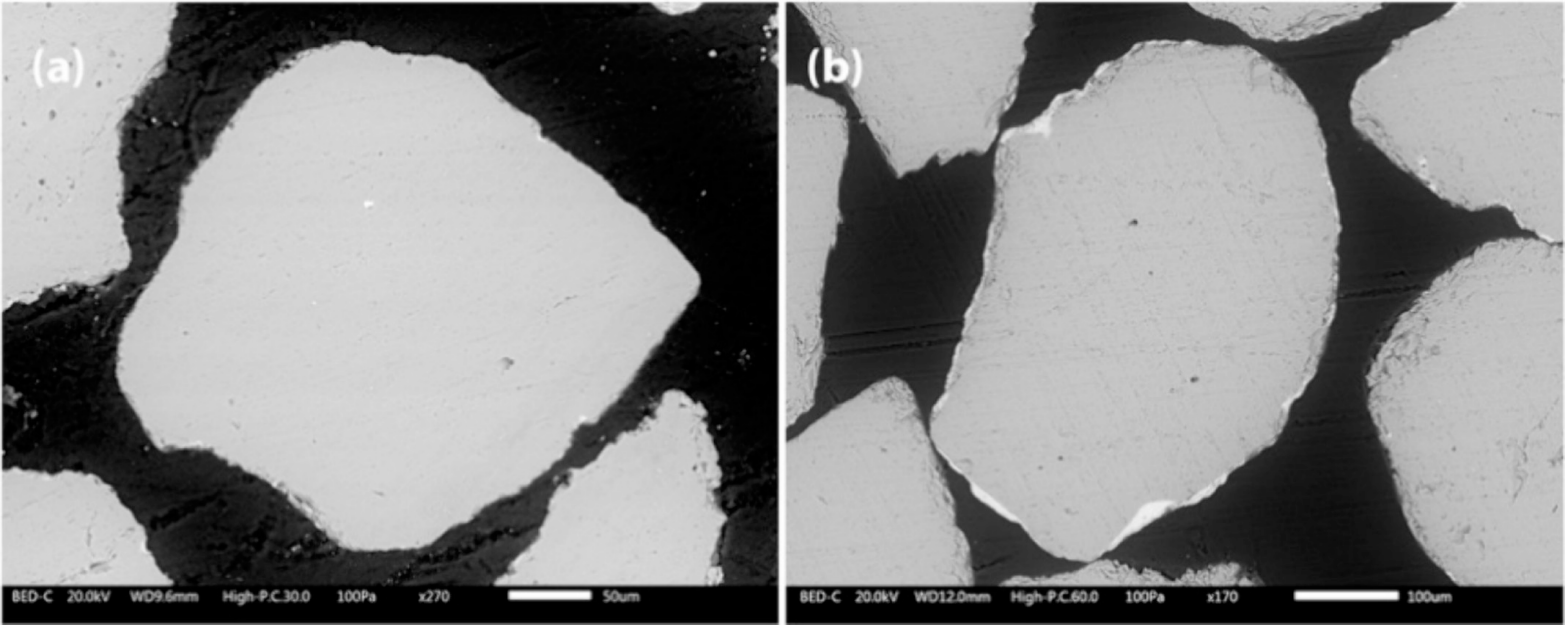
SEM-BSE images of the cross sections of the typical quartz bed particles taken at the end of the campaign from the pilot-scale (96 h) (a) and industrial-scale (∼1000 h) (b) processes.

**Figure 9:**
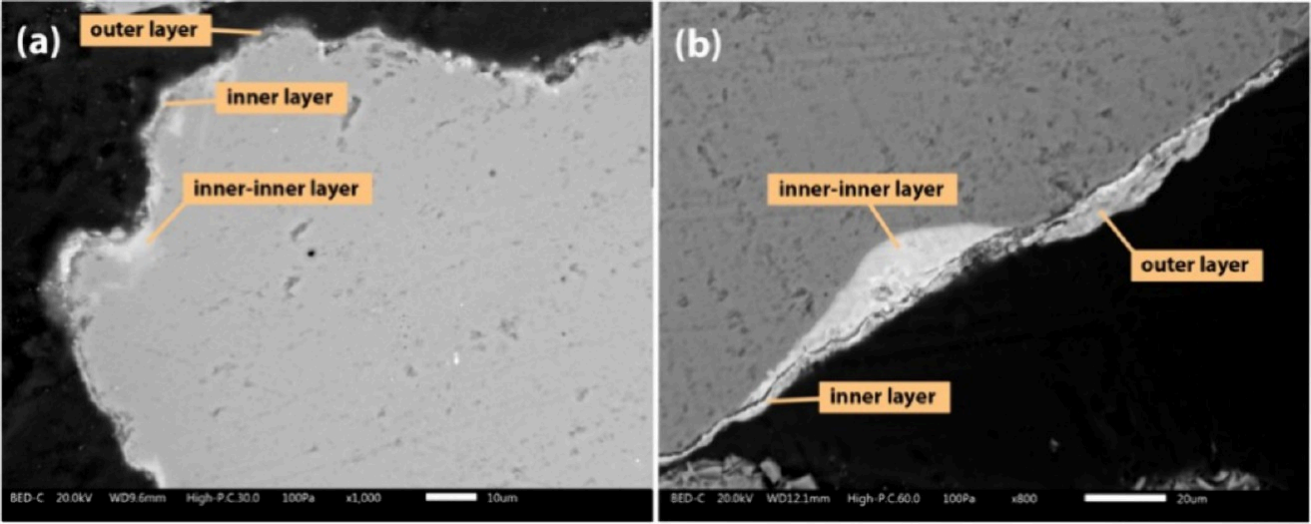
SEM-BSE images of the bed particle layers formed on the typical quartz bed particles taken at the end of the campaign from the pilot-scale (96 h) (a) and industrial-scale (∼1000 h) (b) processes.

The outer layer exhibited a composition similar to that of fuel ash, similar to previous observations in related studies.^10,29,41,43^ The elemental composition of the inner layer and inner-inner layer (Figure 10) was also consistent with previous findings in the literature.^10^ The inner layer was primarily composed of Si, Ca, and Mg (except O), with the outer layer exhibiting higher concentrations of Ca and Mg than the inner layer. In contrast, the inner-inner layer was predominantly composed of Si and K. Although the presence and thickness of these layers varied between convex and concave regions, previous findings,^10^ as well as our current observations, have shown that their chemical composition remains consistent across different surface geometries.

**Figure 10:**
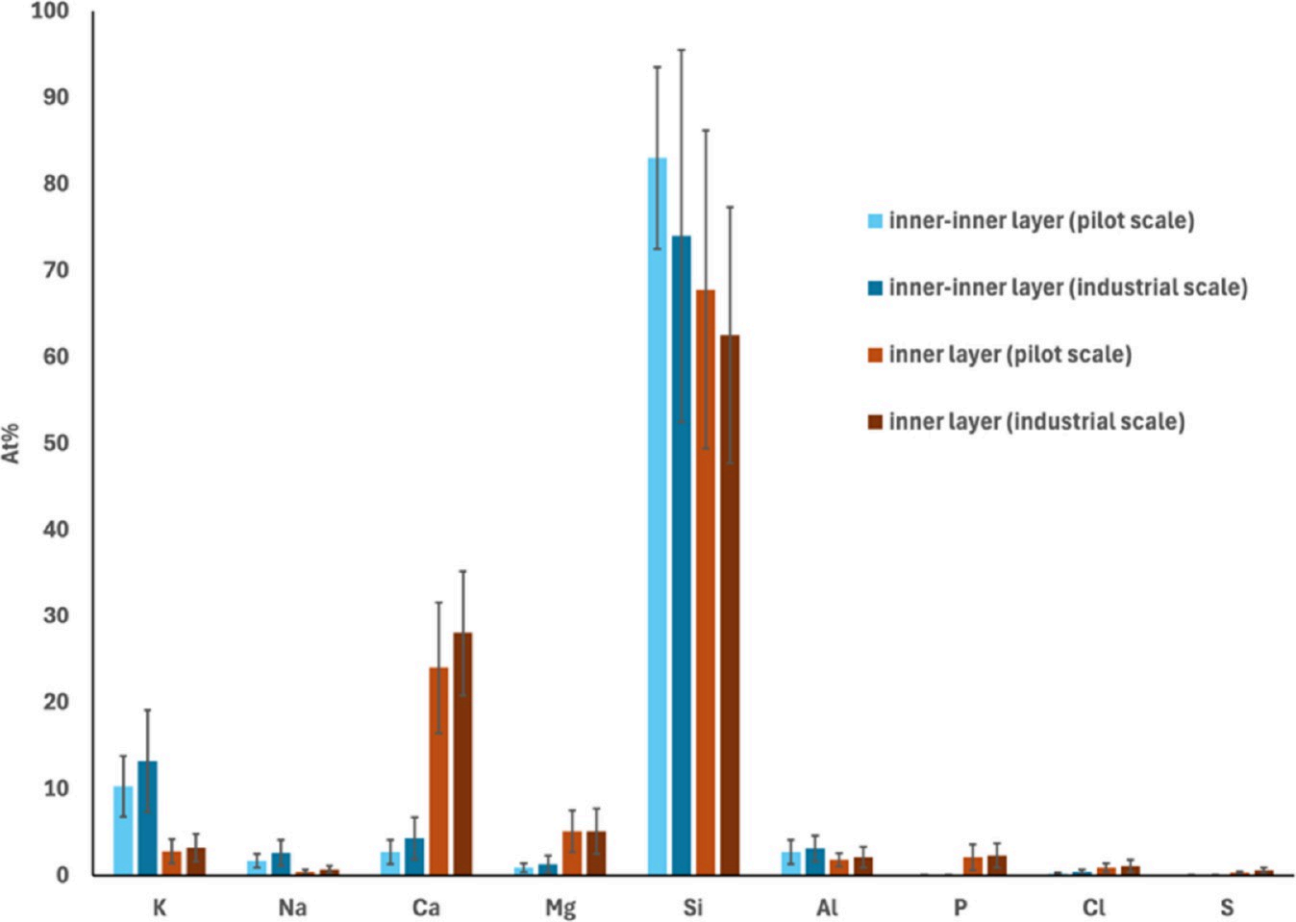
Average elemental composition (on a C- and O-free basis) of the inner layer and the inner-inner layer found on quartz bed particle samples collected from the pilot-scale (96 h) and industrial-scale (∼1000 h) processes. The error bars show the standard deviation at a 95% confidence interval.

### 3.2 Bed Particle Layer Thickness

Figure 11 illustrates the average bed particle layer thickness measured at various time intervals during the bench-scale process, as well as at the end of the campaign for both the pilot-scale (96 h) and industrial-scale (∼1000 h) processes. This provides an overview of how the bed particle layer evolves over time, covering both the early stages and extended exposure periods. It is important to note that the bed particle layer was initially very thin and only sporadically present on certain convex areas, making it impossible to measure its thickness for the samples taken after 4 and 8 h from the bench-scale process. Additionally, for the samples taken after 20 and 22 h, the precise boundary between the inner and outer layers could not be clearly defined, preventing an accurate measurement of their respective thicknesses. Therefore, the values presented in Figure 11 for these samples should be interpreted as the total bed particle layer thickness.

**Figure 11:**
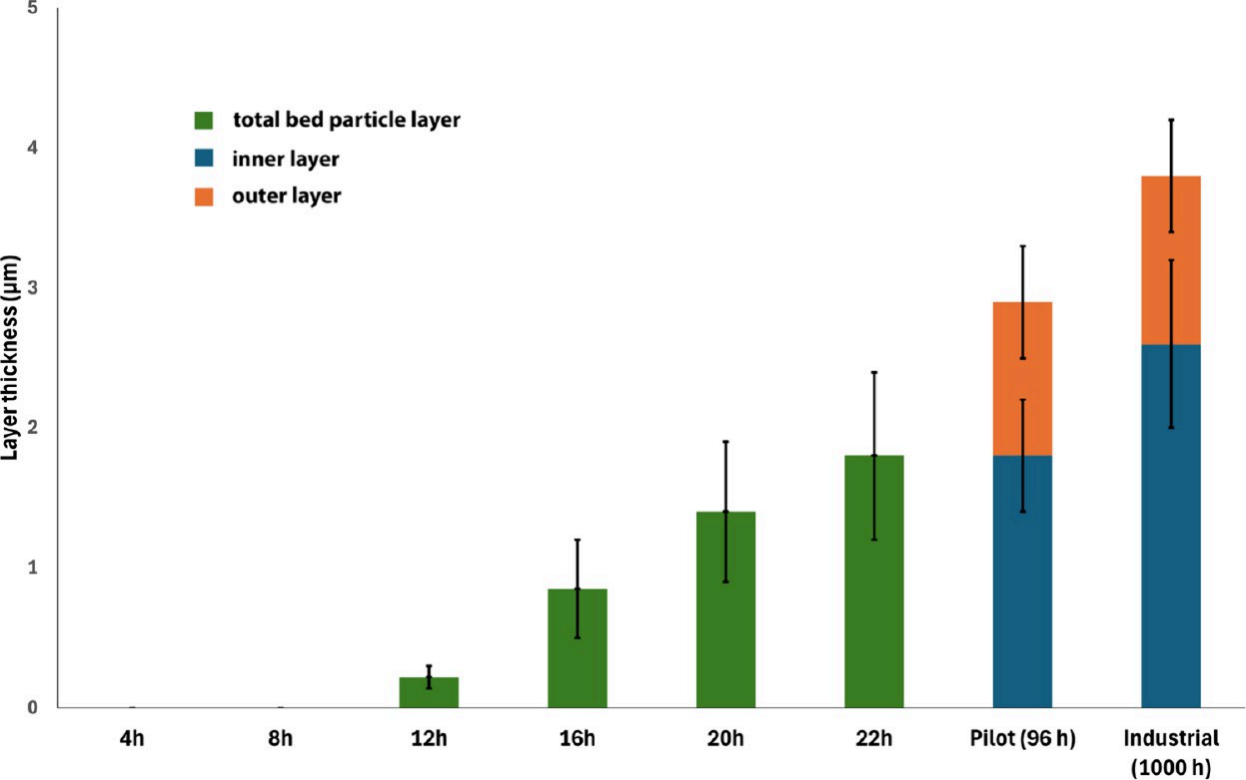
Average bed particle layer thickness for typical quartz bed particle samples collected at different time intervals from the bench-scale process and at the end of the campaign from the pilot-scale (96 h) and industrial-scale (∼1000 h) processes. Thickness values represent the mean of coated regions only; uncoated (zero-thickness) areas were not included. The error bars show the standard deviation at a 95% confidence interval.

The general trend observed is that the bed particle layer thickness increases over time, stabilizing after a certain thickness is reached during longer exposures. Although the deposition of Ca-rich fuel-derived ash particles and likely inward diffusion of Ca into the bed particle core continue throughout the process, the growth rate of the bed particle layer decreases over time, likely due to a balance being reached between the rates of layer formation and attrition. SEM observations further revealed that layer formation was highly nonuniform during the early stages, with deposits predominantly found on convex surfaces, while concave regions often remained uncoated. The limited layer growth observed in this study is consistent with the strongly diffusion-limited nature of Ca^2^^+^ incorporation into quartz surfaces at pyrolysis-relevant temperatures. Reported Ca^2^^+^diffusion coefficients in silicate or alkali-modified amorphous matrices are typically on the order of 10^–^^19^ to 10^–^^18^ m^2^ s^–^^1^ at ∼650 °C, which places fast pyrolysis operation firmly in the slow solid-state diffusion regime. In contrast, at temperatures characteristic of fluidized-bed combustion (≥800 °C), Ca^2^^+^ diffusivities increase to approximately 10^–^^16^ to 10^–^^15^ m^2^ s^–^^1^, representing a 2–3 order of magnitude acceleration.^52,53^ This substantial increase in Ca mobility at higher temperatures suggests why combustion systems exhibit continuous growth of Ca-rich inner layers, whereas the layers formed under fast pyrolysis remain thin (<4 μm) and their growth rate decreases markedly after the first 12–24 h. These quantitative considerations support the conclusion that layer development in fast pyrolysis is governed by slow solid-state diffusion, in contrast to that under combustion conditions.

An independent two-sample *t* test showed that the inner layer thickness increased significantly between the pilot-scale and industrial-scale samples (*p* < 0.05), which is consistent with the continued inward diffusion of Ca over time. In contrast, the outer layer did not exhibit a statistically significant change, likely due to a balance between ash deposition and attrition at the surface.

While attrition primarily impacts the outer layer, its effect is not limited to this region. Since the outer layer serves as the source of Ca for diffusion into the bed particle core and the subsequent growth of the inner layer, attrition indirectly influences both layers. However, the impact on the outer layer is more pronounced, as it governs the Ca supply necessary for the inner layer’s development. Additionally, the extended length of the inner layer may increase the diffusion distance for Ca, which could further contribute to the reduction in the growth rate.

Surface SEM images (Figure 4) also reveal a distinct difference in the attrition of bed particles between the pilot-scale and industrial-scale processes. The bed particles collected from the pilot-scale process retained a greater amount of fine fuel-derived ash particles on their surfaces. In contrast, the bed particles from the industrial-scale process exhibited smoother surfaces, with fewer fine ash particles. This difference might be attributed to the shorter exposure times in the pilot-scale process, which likely resulted in a lower degree of sintering. Meanwhile, in the industrial-scale process, the longer exposure times facilitated sintering, leading to the smoother surfaces observed in the SEM images.

### 3.3 Suggested Time-Dependent Bed Particle Layer Formation Process

At the operating temperature of the pyrolysis reactor (500 °C), a significant portion of Cl is expected to be released in the gas phase as HCl.^54–57^ A major portion of sulfur is also released at this temperature due to the decomposition of organically bound sulfur.^57,58^ In contrast, Ca is found in the remaining char as Ca-oxalates and Ca-carbonates formed by thermal decomposition of Ca-oxalates.^59,60^ Mg is also likely to be retained in the char, as it typically follows a transformation trend similar to that of Ca during ash transformation processes.^61^ It has been shown that K also exhibits a very high retention in the solid phase during pyrolysis at lower temperatures (<700 °C), where it is either organically bound to the carboxyl groups in the char or present as solid K-salts, such as KCl.^54,58^ Therefore, since a major portion of ash-derived Ca, Mg, and K is retained in the char and given the relatively low temperature, the extent of interaction with bed particles in the pyrolysis reactor is expected to be limited.

In the fluidized-bed combustor (∼650 °C) Ca, K, and Mg will be released during the char combustion likely as carbonates/mixed carbonates.^60^ In the splash zone above the bubbling dense bed, char particles can experience elevated temperatures^10^ and in this zone the CaCO_3_ can be converted to CaO and part of the K found in the carbonates/mixed carbonates can be released to the gas phase and found as KOH.^57^

That said, the formation of bed particle layers is highly unlikely in the pyrolysis reactor due to the relatively low operating temperatures and limited ash–bed interaction. Instead, this phenomenon is primarily initiated in the fluidized-bed combustor, where elevated temperatures and increased ash mobility promote the deposition and reaction of the fuel-derived ash compounds with the bed material. The bed layer formation is thus suggested to begin in the early stages of the process through the deposition of mainly Ca-rich fuel-derived ash particles (probably CaCO_3_), primarily onto the convex regions of the quartz bed particle surfaces. This is followed by the diffusion of Ca^2^^+^ from these surface deposits toward the quartz core, where they likely react to form Ca-silicate. This reaction results in the development of the inner layer, which can thus be termed a reaction layer. It is also theoretically possible that during the early stages of the process, gaseous alkali species interact directly with the quartz surface in the splash zone. However, due to the short residence time of the bed particles in this zone and the time-dependent nature of the reaction, it may not become sufficiently developed to be detected on a significant number of bed particles within the detection limits of the analytical approach applied in this study.

Over time, the inner layer becomes more established and uniform across the convex areas as the continuous inward diffusion of Ca^2^^+^ leads to increased Ca enrichment. In concave areas, the formation of the inner layer is delayed compared to the convex regions, consistently resulting in thinner layers.

As evidenced by SEM observations, a structurally consolidated outer layer begins to form after approximately 20 h into the process. Formation of the outer layer is driven by continued deposition of Ca-rich fuel-derived ash particles onto the bed particle surface, again predominantly in convex regions. Since this layer is primarily a product of fuel-derived ash particle deposition, its composition closely resembles that of the bed ash, dominated by Ca.

At extended exposure durations (i.e., several days), an additional layer dominated by Si and K, termed the inner-inner layer, emerges. In previous studies on combustion, the formation of this layer has been attributed to the penetration of gaseous K into the bed particle core through areas not fully covered by the bed particle layer or through cracks in the existing inner layer.^41,43^ Once penetrated, K can react with the bed material to form K-silicates. As mentioned earlier, in the fast pyrolysis process, gaseous K is likely present in the splash zone of the combustion reactor, which can explain the formation of the observed inner-inner layer during long exposure. Notably, the inner-inner layer was primarily found in concave areas where the outer layer was absent and the inner layer was either absent or very thin, allowing gaseous alkali species to directly interact with and diffuse into the quartz core.

While the formation of both the inner and outer layers is continuous over time, the total bed particle layer thickness tends to converge to a certain limit during long exposures. This stabilization is attributed to attrition processes, which predominantly affect the outer layer, and to a reduced diffusion rate of Ca^2^^+^ into the inner layer due to the increasing diffusion distance. The above-discussed bed particle layer formation process is schematically illustrated in Figure 12.

**Figure 12:**
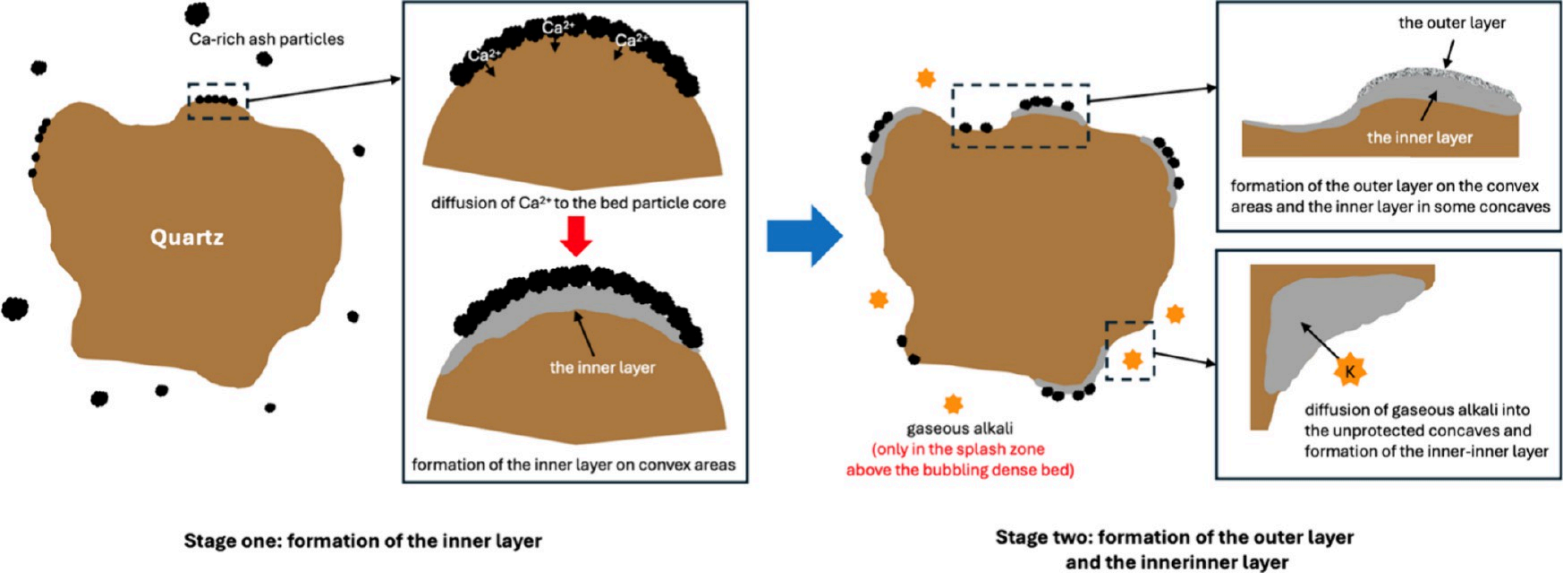
Schematic illustration of the bed particle layer formation process on quartz bed particles utilized in the fast pyrolysis of wood.

### 3.4 Comparison of the Time-Dependent Quartz Bed Particle Layer Formation in Fast Pyrolysis and Combustion of Wood/Woody Biomass

To enable a more accurate comparison, this study and the combustion references 29,41,43,50 employed similar fuel types and quartz bed materials, with nearly identical ash-to-bed material ratios, allowing the focus to remain on the process itself. The combustion cases included one bubbling fluidized-bed (BFB) system operated at 800–880 °C using a softwood mix as fuel over a 23-day period with a bed exchange rate of approximately 3% per day and one circulating fluidized-bed (CFB) system operated at 780–850 °C using sawdust over a 13-day period with a bed exchange rate of about 10% per day. Despite differences in fluid dynamics and exposure times, all systems maintained an ash input of approximately 1 g/h per 1 kg of bed material. This consistency in ash exposure enables a systematic comparison of the time-dependent evolution of bed particle layers, while the key difference between fast pyrolysis and fluidized-bed combustion lies in the operating temperature. These temperature differences are known to strongly influence the mechanisms and rates of layer formation, particularly in relation to ash melting, diffusion, and reaction kinetics.^10^

Overall, the time-dependent formation and composition of the bed particle layers on quartz bed particles were observed to be similar in both fast pyrolysis processes and fluidized-bed combustion of woody biomass with the process beginning with the formation of the inner layer primarily in the convex areas of the bed particle surface. Over time, the outer layer also develops on these areas, and with some delay, the bed particle layer begins to form in the concave areas until the entire bed particle surface is covered. The inner-inner layer is also observed mainly in the concave areas of both processes. However, a key difference between the two processes is the lower concentration of Ca in the inner layer and the significantly thinner bed particle layer in fast pyrolysis compared to combustion.

The formation of the bed particle layer begins with the deposition of Ca-rich particles onto the surface of the quartz bed particles, which occurs primarily through impact.^49^ In combustion, the temperature is high enough to release a considerable portion of K into the gas phase, facilitating the reaction of gaseous alkalis with the bed material. This reaction leads to the formation of a sticky melt on the bed particle surface, which enhances the adhesion of the Ca-rich particles upon impact.^41^ However, in the fast pyrolysis process, the gaseous alkali species are more localized in the splash zone of the combustor, where particles pass through for only short periods, thus lowering the adhesion of the Ca-rich particles to the bed particle surface.

Following deposition, the bed particle layer formation process shifts to diffusion, where Ca^2+^ is transported from the deposited bed ash particles into the bed particle core.^41,43^ Temperature plays a crucial role in the diffusion process, as the rate of mass transfer is highly sensitive to temperature.^45^ In fast pyrolysis, which occurs at lower temperatures, the mass diffusivity coefficient is significantly reduced, hindering the inward diffusion of Ca. Theoretically, when the operating temperature is reduced by 100 °C from typical combustion conditions, the mass transfer coefficient of Ca^2+^ into the inner layer (CaSiO_3_) decreases by nearly 2 orders of magnitude.^41,62^ As a result, the slower diffusion limits Ca penetration, yielding a thinner bed particle layer with lower Ca concentrations in the early stages. However, over time, the concentration of Ca (and, to some extent, Mg) in the inner layer gradually increases. Eventually, the Ca:Si ratio approaches an equimolar value, indicating the probable formation of CaSiO_3_ (wollastonite), which has also been reported in previous studies on similar systems. In contrast, fluidized-bed combustion processes operate at significantly higher temperatures, which substantially enhances the mass diffusivity of calcium. This allows Ca to diffuse more rapidly into the bed particles, resulting in the formation of a thicker, more Ca-enriched layer. In such high-temperature environments, the Ca:Si ratio in the inner layer often exceeds unity over time, favoring the formation of calcium silicates with a higher calcium content (i.e., Ca_2_SiO_4_ or potentially Ca_3_SiO_5_).^41^ The coexistence of different Ca-silicates with distinct crystal structures can introduce lattice mismatches. This structural incompatibility is believed to contribute to developing internal cracks within the inner layer, particularly in aged bed particles retrieved from combustion.^29^ In contrast, in fast pyrolysis, the lower diffusivity and eventual stabilization at an equimolar Ca:Si ratio result in a more uniform and stable inner layer without any cracks, even after prolonged exposure times exceeding 1000 h. This difference further underscores the role of thermal conditions in governing the physicochemical interactions between the bed particles and ash-forming elements.

Another important distinction between fluidized-bed combustion of woody biomass and fast pyrolysis of wood lies in the long-term interaction between alkali species and quartz bed particles. In combustion processes, the inward diffusion of K into the quartz core can lead to the formation of crack layers, features that gradually develop within the particle structure. Over time, these crack layers can grow and coalesce, eventually bridging across the particle and leading to mechanical weakening. After approximately 2 weeks of exposure, this process may result in the fragmentation of bed particles into finer fragments.^29^ In contrast, such degradation phenomena were not observed in the present study, even after extended exposure times of up to 1000 h under fast pyrolysis conditions. The absence of the crack layers under fast pyrolysis conditions can primarily be attributed to the lower operating temperature, which significantly limits the diffusivity and reactivity of gaseous alkali species and the bed material. In addition, unlike in combustion, where gaseous alkalis can reach the particle core through cracks in the inner layer (even when a bed particle layer is present), such cracks are missing in the pyrolysis environment. This likely enhances the protective function of the inner layer and further restricts alkali–bed interactions.

From an operational viewpoint, these mechanistic differences have direct implications for long-term reactor performance and bed material management in fast pyrolysis systems. The slow and diffusion-limited growth of Ca-rich layers at ∼650 °C, combined with the absence of crack layer formation and particle fragmentation, indicates that quartz remains structurally stable under a prolonged fast pyrolysis operation. This contrasts with combustion conditions, where enhanced alkali diffusivity and the formation of Ca-rich polymorphs (e.g., Ca_2_SiO_4_ and Ca_3_SiO_5_) accelerate internal cracking and ultimately promote particle disintegration.

The stability of quartz under pyrolysis conditions suggests a low risk of temperature-driven agglomeration or a loss of mechanical integrity, supporting its suitability as a long-term bed material in continuous fast pyrolysis operation. However, the gradual enrichment of Ca (and to a lesser extent Mg) within the inner layer observed in both scales may still influence the catalytic behavior of the bed over extended campaigns. Even in the absence of melt formation, Ca-rich reaction layers may facilitate secondary cracking or deoxygenation reactions, potentially affecting the FPBO yield and selectivity.

## 4 Conclusions

The time-dependent formation of bed particle layers on quartz during the fast pyrolysis of wood was systematically investigated across bench-, pilot-, and industrial-scale systems. The results reveal distinct temporal, morphological, and compositional trends that clarify the mechanisms governing ash–quartz interactions under pyrolysis conditions. Together, these observations provide insight into not only fundamental layer formation pathways but also implications for reactor stability, material suitability, and long-term process performance in industrial fast pyrolysis applications. The key findings are summarized as follows:

- Bed particle layer formation begins early in the process, mainly with the deposition of Ca-rich fuel-derived ash particles, mainly on the convex regions of the bed particle surfaces. This leads to the formation of Ca-rich layers (probably Ca-silicates) as a Ca-reaction layer.
- The formation of the inner layer in the concave regions occurs with a delay, and consequently, the layer thickness in these areas remains lower compared to the convex regions of bed particles of the same age.
- The outer layer emerges mainly on the convex surfaces after approximately 1 day of operation and originates from the continued deposition of the fuel-derived ash particles.
- The formation of a K–Si-rich inner-inner layer is observed around the same time as the outer layer, primarily in connection with the concave regions. This is likely due to the inward diffusion of gaseous alkali species.
- The growth of the bed particle layer decreases over time, likely due to a reduced growth rate of the inner layer, which may be caused by the increasing diffusion distance.
- Despite the time-dependent formation and composition of the bed particle layers on quartz bed particles being similar in both fast pyrolysis and fluidized-bed combustion processes, the thickness and Ca concentration of the inner layer were consistently lower in the fast pyrolysis samples. This is likely due to the lower operating temperatures in the fast pyrolysis process.
- The extent of inner-inner layer formation and K concentration within it are lower in fast pyrolysis compared to combustion. Moreover, crack layers are absent within the resolution limits of the SEM analysis used in this study. These differences can largely be attributed to the lower operating temperatures in fast pyrolysis. Specifically, the absence of cracks in the inner layer may further limit the penetration of gaseous species, contributing to the lack of crack layer formation.

## ⊥

Present address: BEST Biogas B.V., Achterom 4, 7591 BA Denekamp, The Netherlands
